# P276: Impact of nosocomial infection prevention program at departmental hospital center of Zou Collines, Benin

**DOI:** 10.1186/2047-2994-2-S1-P276

**Published:** 2013-06-20

**Authors:** TA Ahoyo, JM Agoi, A Attolou Gbohoun

**Affiliations:** 1 Health Minstry of Benin, Cotonou, Benin

## Objectives

The study was performed to determine the impact of hygiene measure promotion on the frequency of nosocomial infection (NI).

## Methods

From April 2004 for a period of six years (2004-2010), we conducted an annual one day prevalence study of NI, preceded by a campaign of hygiene measure promotion. In the absence of national NI definition, we used the protocol drawn up in 2002 by CLIN Sud-Ouest (France). Patient’s characteristics were recorded and data collection was performed by nurses who had been trained. Quick audit was applied to assess the hand hygiene compliance.

## Results

For the six year period 484, 629, 688, 750, 780, 1115 patients were included, resulting in a prevalence of NI of 41%, 31%, 22%, 18%, 13%, and 13% in 2004 to 2010 respectively, corresponding in absolute reduction of 28% of IN (p < 10^–5^). Compliance of hand hygiene was respectively 23%, 43%, 45%, 47%, 53%, 55%. The increase of hand disinfection of 30% (p < 10^-5^) was noticed because of the use of alcohol solution. The demographics data were almost identical in all six year. Urinary infections were the most prevalent; a reduction of 15% was noticed among infection related to catheter.

## Conclusion

These findings suggest that hand washing should become an educational priority in our context of limited budgets; appropriate allocution of resources is needed.

## Competing interests

None declared

